# Complicated placement of a nasogastric tube in the gastric mucosa: A case report and literature review

**DOI:** 10.1111/nicc.13178

**Published:** 2024-10-11

**Authors:** Yiqi Zhang, Yuzhi Gao, Linyan Zeng, Juan Hu, Xia Zheng

**Affiliations:** ^1^ Intensive Care Unit, The First Affiliated Hospital Zhejiang University School of Medicine Hangzhou P. R. China

**Keywords:** nasogastric tube, nutrition‐nasogastric tube complications, submucosal migration

## Abstract

Nasogastric tubes (NGTs) are commonly used in the intensive care unit (ICU) and are often inserted blindly at the bedside. Previous studies have highlighted various complications associated with NGT misplacement, including epistaxis, pneumothorax and even fatal perforations. To reduce the incidence of complications, guidelines recommend confirming the correct position of the NGT through radiography, pH testing, end‐expiratory carbon dioxide monitoring, ultrasonography, etc. Herein, we present the case of a 78‐year‐old man who experienced sudden dyspnoea, was brought to the ICU and subsequently developed gastrointestinal bleeding following improper NGT placement. In this patient, air was rapidly injected down the NGT while auscultating for a ‘whooshing sound’ over the epigastrium. However, the correct position of the NGT was eventually confirmed by X‐ray. Urgent upper gastrointestinal endoscopy revealed an oesophagogastric submucosal tunnelling of the NGT. This case emphasizes that auscultation may be unreliable and no longer encouraged. Additionally, various verification methods may not detect such rare complications related to NGT placement, making it necessary to focus on the emerging clinical manifestations following NGT insertion. Moreover, gaining further insight into the history of gastrointestinal diseases in patients may be beneficial.

**Relevance to Clinical Practice:**

This case underscores the importance of noting resistance during a blind nasogastric tube (NGT) insertion in the intensive care unit (ICU). Additionally, the ‘whooshing testing’ for tube placement verification is not recommended. Although radiographic confirmation remains the gold standard, it may not effectively identify rare complications. Furthermore, emerging clinical signs (such as the abnormal nature of the gastrointestinal decompression drainage fluid, hypotension and anaemia) after insertion may suggest NGT misalignment. Finally, in urgent ICU settings, the patient's history of gastrointestinal disease should not be overlooked, as it can lead to complications such as gastrointestinal bleeding.

**What is known about the topic**

- Serious complications have been reported after nasogastric tube (NGT) insertion blindly in the intensive care unit (ICU).
- Immediate confirmation of the tube tip position after placement is crucial.
- Current validation methods all have limitations that can lead to the erroneous belief that the NGT is correctly placed.

**What this paper adds**

- Although radiographic validation remains the gold standard, it may not be effective in identifying rare complications.
- Be alert for clinical signs following NGT insertion (e.g. abnormal nature of gastrointestinal decompression drainage fluid, hypotension and anaemia), which may indicate NGT misalignment.
- A patient's history of gastrointestinal disease should not be ignored before NGT insertion in urgent ICU settings.

## 1 BACKGROUND

Nasogastric tube (NGT) insertion is a common procedure performed in the intensive care unit (ICU). Despite being a routine bedside procedure often performed blindly, serious complications such as pneumothorax and aspiration pneumonia have been reported.^1^, ^2^ To avoid complications because of NGT misplacement, immediate confirmation of the tube tip position after placement is crucial. Currently, radiography is considered the gold standard for confirming correct NGT positioning.^3^ This implies that patients in the ICU may require multiple NGT placements or repositioning, which can inadvertently lead to accidental extubation and haemodynamic instability. Another widely recommended bedside method is the aspiration of NGT contents for pH testing.^4^, ^5^, ^6^ Epigastric auscultation (the ‘whooshing test’) is commonly performed in our ICU, although numerous studies have identified it as an unreliable traditional technique for determining proper NGT placement.^7^, ^8^ However, no optimal validation method currently exists, and each method has certain limitations (Table 1), which can lead to the erroneous belief that the NGT is correctly placed.^9^

**TABLE 1:** Advantages and disadvantages of nasogastric tube placement verification.

Tip position verification	Specific method	Advantages	Disadvantages
The ‘whooshing test’ or air insufflation method	Rapidly injected air down the NGT while auscultating ‘whooshing sound’ over the epigastrium	Simple, convenient and widely used^10^, ^11^	This method remains controversial and is no longer recommended. Gas injected into the lungs or trachea may produce a similar sound. The lack of specificity can lead to confusion.^12^
pH testing	To confirm the location of the NGT, pH testing of its aspirate was performed as a first‐line method. A pH of ≤5.5 indicates that the NGT is correctly placed in the stomach, while a pH of ≥6 may indicate placement in the gut or respiratory tract.^13^	Fast and convenient	The pH of the gastric fluid aspirate can increase to 6 or higher by antacids and acid inhibitors. Colorimetric test strips require subjective interpretation, posing challenges for accurate readings. No aspirate can be obtained.^14^
X‐ray	X‐ray examination^15^	X‐ray is the gold standard for distinguishing between gastric and pulmonary placement of an NGT.^15^	Misreading the X‐ray. Excessive radiation. X‐rays are not readily available in nursing homes, rehabilitation centres and home care settings^16^ as well as in the ICU.^17^
End‐tidal carbon dioxide monitoring	Connect the instrument directly to the end of the NGT tube. If the pressure reading is ≥15 mmHg, the NGT may have entered the airway. Conversely, a pressure reading of ≥10 mmHg indicates that the NGT may not be in the airway.^18^	The presence of NGT in the stomach or airway was confirmed using quantifiable indicators.	Detecting instruments in ordinary wards can be challenging. Only NGT placed in the airway can be detected.^19^, ^20^
Bedside abdominal ultrasound	NGT was detected in the upper abdominal gastric region using an ultrasound probe.^16^	Ultrasound can directly and clearly visualize the presence or absence of an NGT in the stomach. This technique is straightforward for ICU medical staff to understand.^21^, ^22^	Identifying the entire tube from the nose to the gastrointestinal tract is challenging. Two operators are needed. Technical difficulties arise in obese patients, patients who undergo laparotomy and patients with an open abdomen, abdominal wall defect or drainage.^23^, ^24^
A single‐use, small‐bore nasogastric feeding tube with a miniature camera embedded in the distal end	An indwelling NGT directly placed using camera navigation^16^	Clear images of the stomach were obtained, which allowed for direct observation and prevented malposition.^25^, ^26^	Trained clinicians are required to accurately identify the anatomical landmarks of the oesophagus, trachea or stomach. Additionally, they must consider the potential discomfort from passing the camera tip through the patient's nose and the high cost of the device.^24^, ^27^
Electromagnetic‐guided postpyloric feeding tube placement	The feeding tube's electromagnetic emitter at the tip was used to monitor the tube's trajectory during placement through the receiver device and display placed outside the body. The position of the tip of the feeding tube was determined in real time.^28^	Lung misplacement or gastric twisting can be easily detected by the system, allowing the operator to make timely adjustments and reduce the risk of X‐ray radiation.^28^	Proficient clinicians are required.^29^

Abbreviations: ICU, intensive care unit; NGT, nasogastric tube.

Herein, we report a case demonstrating that the validation methods, including radiographic confirmation, are often ineffective in identifying rare complications associated with NGT placement; however, using the most reliable method can prevent the occurrence of most complications. Additionally, close attention to the special clinical manifestations following NGT insertion revealed a misalignment. This case underscores the importance of not only recognizing the limitations of current validation methods but also paying special attention to clinical symptoms or abnormal laboratory results that may suggest NGT misplacement. In the ICU, clinicians should be mindful of a patient's history of any upper gastrointestinal disease, which can be easily overlooked.

## 2 CASE ANALYSIS

**FIGURE 1:**
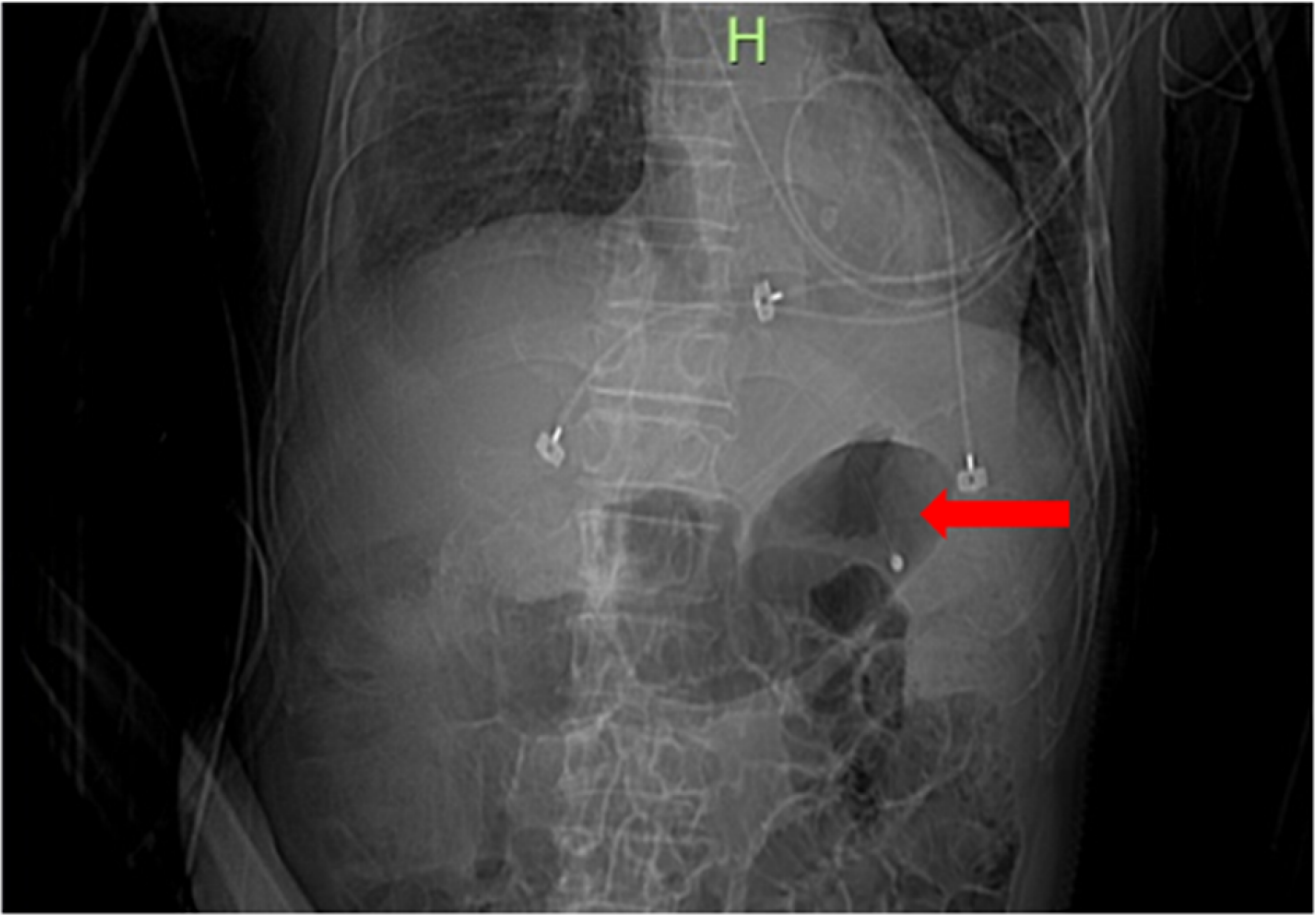
X‐ray image obtained immediately after tube placement, demonstrating the correct insertion of the nasogastric tube into the stomach.

During the insertion of an NGT (polyvinyl chloride transnasal feeding tube, radiopaque with a guide wire, unweighted tip, an outer diameter of 4.7 mm and an inner diameter of 3.2 mm; Baitong tube, Lingze Medical, Beijing, China), significant resistance was felt at 55 cm. Therefore, the insertion was terminated and the NGT was not removed. Air was rapidly injected down the NGT while auscultating the epigastrium, but no ‘whooshing sound’ was heard so that we removed the NGT. Then, the NGT insertion was repeated immediately, but persistent resistance was encountered. The patient presented with epistaxis because of friction of the NGT, and we replaced the NGT with a thinner NGT (polyurethane transnasal feeding tube, radiopaque with a guide wire, unweighted tip, an outer diameter of 4.5 mm and an inner diameter of 3.5 mm; Freka tube, Fresenius Kabi AG, Miekinia, Poland), which had a rounded bolus tip.^30^ During the insertion of the new NGT, a slight resistance was noted at 55 cm; however, NGT placement was completed at 60 cm. Although the fluid aspirated from the stomach showed hemic, the ‘whooshing sound’ was auscultated after air insufflation. X‐ray imaging (Figure 1) confirmed that the NGT was appropriately positioned.

**FIGURE 2:**
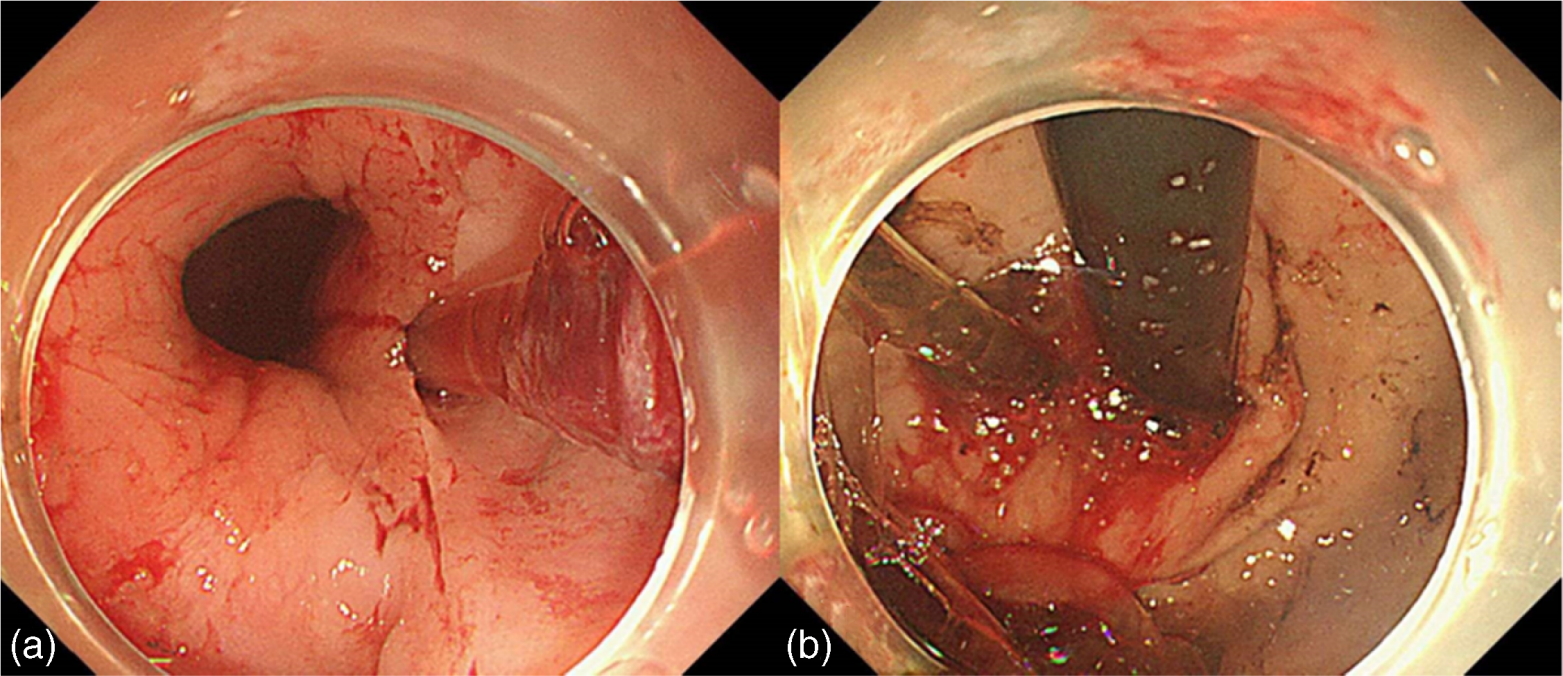
(a) An image captured during gastroscopy revealing the direct insertion of the nasogastric tube (NGT) under the cardia mucosa. (b) An image captured during gastroscopy showing the NGT re‐emerging from the gastric submucosa.

**FIGURE 3:**
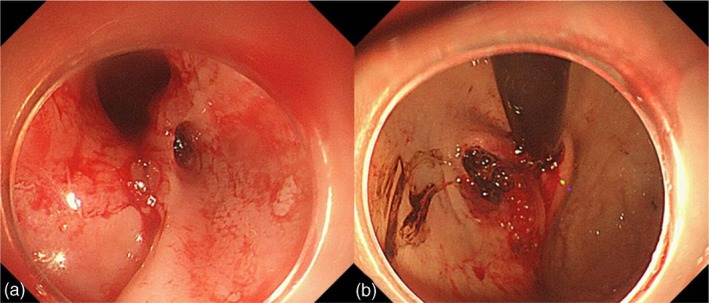
Images captured during gastroscopy showing the entrance (a) and exit (b) of the ‘tunnel’ after removing the nasogastric tube.

Intermittent drainage of bloody fluids from the NGT was observed. However, his blood pressure remained stable, while his haemoglobin level fluctuated between 63 and 65 g/L. Given the presence of epistaxis, an otolaryngologist performed a bedside nasal endoscopy, which revealed the absence of bleeding spots. The following day, hemic fluid was aspirated from the stomach, the haemoglobin level decreased by approximately 15% and the blood pressure remained stable. Emergency gastroscopy suggested a potential gastric perforation because of the NGT. Chest and abdominal computed tomography (CT) examinations were immediately performed to determine the need for emergency surgery. Surprisingly, no evidence of perforation was observed on the CT scans. Repeat gastroscopy suggested that the NGT traversed from the mucosa of the cardia (Figure 2a) to another part of the gastric mucosa (Figure 2b). The NGT was extracted under gastroscopic guidance, which revealed the mucosal tunnel entrance and exit (Figure 3a,b). As no active bleeding was observed, a new NGT was placed under gastroscopic guidance.

**FIGURE 4:**
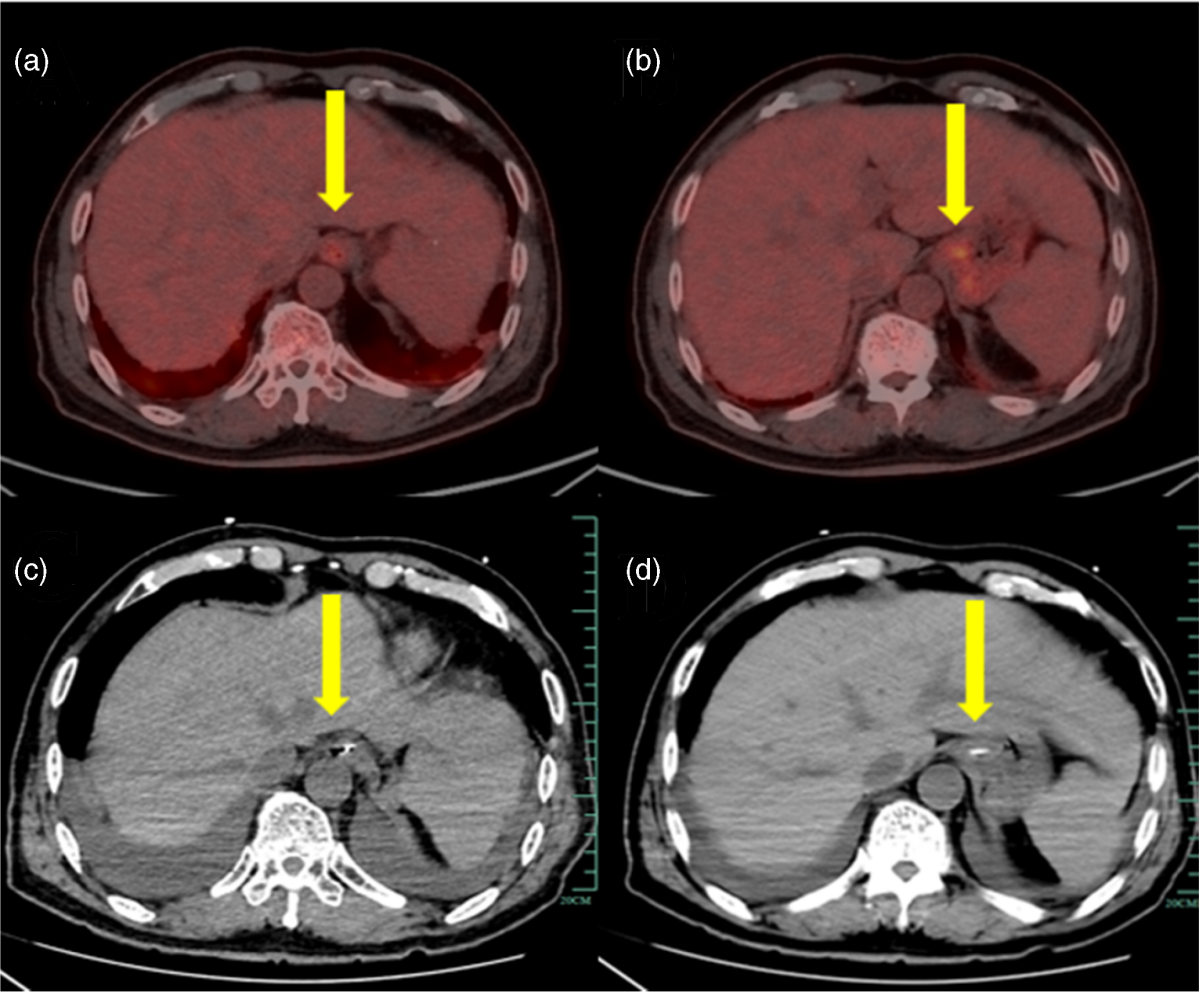
(a, b) Positron emission tomography‐computed tomography images showing increased fluorodeoxyglucose metabolism around the cardia, suggestive of inflammatory lesions. (c, d) Abdominal computed tomography images indicating the indwelling of the gastric tube at the cardia in the digestive tract lumen, with mucosal discontinuity.

Although our patient reported the absence of discomfort in the gastric region, a positron emission tomography‐CT (PET‐CT) examination conducted 1 month earlier revealed increased fluorodeoxyglucose metabolism in the lower oesophagus near the cardia (Figure 4a), indicating an inflammatory lesion. An urgent abdominal CT was performed and showed a mucosal discontinuity around the cardia (Figure 4b), coinciding with the location of the PET‐CT lesion. The lesions at the same location may explain the presence of an inflammatory lesion around the cardia at the base of the patient, which is more likely to be damaged by NGT.

Subsequently, the patient fasted for 2 days and was treated with antacids. The drainage fluid from the NGT returned to its normal colour, the haemoglobin level stabilized and enteral nutrition was initiated. Subsequently, he was successfully transferred from the ICU and resumed an oral diet without experiencing epigastric pain, haematemesis or melena.

## 3 A DISCUSSION OF THE CASE OUTCOME

NGTs are commonly used in medical settings, with an annual application being 1.2 million in the United States^31^ and over 1 million in the United Kingdom.^32^ Generally, an NGT is inserted for various diagnostic and treatment purposes^33^ but primarily for nutritional support.^34^ Other uses of NGTs include drug therapy and gastrointestinal decompression, particularly in abdominal surgery.^35^ In the ICU, patients present with a diverse range of complex diseases, often requiring an artificial airway and precluding oral intake because of illness or decreased level of consciousness. Consequently, indwelling NGTs are commonly required in ICUs,^36^ making NGT a standard method for enteral feeding.^16^

NGTs are predominantly placed ‘blindly’ in ICUs, meaning their positions are not confirmed during insertion. The risk of misplacement is high because of the presence of artificial airways, drug sedation and coma, and unconsciousness diminishes clinical warning signs such as pain or coughing. The incorrect placement of NGTs has resulted in approximately 1200–3600 deaths in the United States annually.^31^, ^37^ In a study conducted in an ICU, 740 feeding tubes were inserted. Of these tubes, 14 were mistakenly placed in the tracheopulmonary system, and two patients died from complications directly related to feeding tube placement.^38^ Table 2 summarizes the numerous complications that can arise from NGT placement.

**TABLE 2:** Complications of nasogastric tube placement.

Nasopharynx	Epistaxis, sinusitis, vocal cord paralysis and laryngeal harm, nasogastric tube syndrome, nasopharyngeal discomfort, nasal septal ulcers and pressure injury related to fixation^39^, ^40^, ^41^, ^42^, ^43^, ^44^
Oesophagus	Oesophageal perforation and stenosis and tunnel under the mucosa down to the oesophagogastric junction^40^, ^45^
Stomach and intestine	Gastrointestinal ulcer, gastric perforation and intestinal perforation^1^, ^46^
Respiratory complications	A nasogastric tube that coiled around an endotracheal tube, pneumothorax, pleural effusion, bronchoaspiration related to enteral nutrition, atelectasis, pneumonia and tracheobronchial perforation^1^, ^41^, ^47^
Misconnections	Chemical burn because of the extravasation of gastric juice after accidental tube disconnection Inadvertent connection of the enteral cable set to the central venous catheter, causing oral medications to enter the bloodstream Gastric perforation because of the misconnection of the tube to the oxygen flow meter, resulting in a high oxygen flow in the stomach^48^, ^49^, ^50^
Central nervous system	Intracranial haemorrhage and intracranial perforation^51^, ^52^, ^53^
Others	Cervical vessel perforation, parotitis, abscess, sepsis and hepatic portal vein gas^54^, ^55^, ^56^

Underestimating the risk of NGT mispositioning in clinical practice may have fatal consequences. Therefore, confirming the NGT position after insertion is crucial. Various guidelines and studies have described different methods of verifying NGT placement. We reviewed the previous literature^16^ and summarized the advantages and disadvantages of each approach in Table 1.

### 3.1 Limitations of the conventional methods of NGT insertion tip verification

In this case, the initial auscultation of the upper abdomen was followed by radiography to ascertain the correct positioning of the NGT. The X‐ray showed that the NGT was in the gastric region, but it has been inserted into the gastric mucosa. Similar cases have been documented previously. For example, in a study conducted by Nejo et al. in 2016, two patients were prescribed enteral nutrition following NGT placement, confirmed by auscultating for a ‘whooshing sound’ in the epigastric region and using X‐ray.^54^ One patient presented with high fever and abdominal distension. The CT revealed a significant accumulation of abnormal fluid in the submucosal space of the stomach. Emergency gastroscopy showed that the NGT had entered the submucosal layer of the oesophagus before returning to the gastric lumen. Another patient experienced cold sweats, pale skin and bleeding from gastrointestinal decompression approximately 4 h after enteral nutrition infusion. Emergency gastroscopy revealed that the tip of the gastric tube was inserted into the gastric wall without perforation and showed diffuse gastric submucosal emphysema. Both patients developed life‐threatening intrahepatic portal vein gas (HPVG). If the tip of the NGT or the side hole is in the gastric lumen, a ‘whooshing sound’ may be heard during auscultation, and radiography may indicate correct placement in the stomach. However, endoscopy can show that part of the tube has already penetrated the gastric mucosa, posing a significant risk in clinical practice and may lead to haemorrhage, HPVG, shock or even death.^45^, ^54^

Our case study used the ‘whooshing test’; however, guidelines do not recommend the use of auscultation alone to determine the NGT placement^57^, ^58^, ^59^ because of numerous reports of harmful complications, including death.^12^, ^60^, ^61^, ^62^ A previous case study highlighted these risks.^51^ A patient who underwent resection of a pituitary adenoma had an NGT inserted by two nurses. Air insufflation and auscultation were performed to verify the tube placement. Both nurses believed they heard a ‘whooshing sound’. Subsequently, the patient experienced acute intracranial haemorrhage and pneumocephalus because of the intracranial placement of the NGT through the sphenoid sinus. Auscultation lacks specificity, and gas injected into the lungs or trachea may produce a similar sound.^12^ Additionally, existing guidelines recommend X‐rays as the gold standard for verifying NGT position and advocate for pH testing to quickly confirm placement.^5^, ^6^, ^61^ Unfortunately, pH testing was not employed in our patient, highlighting an area for improvement. For the complication we reported, the result of pH test might not be suggestive because the tip of the NGT was in the stomach cavity.

### 3.2 Focus on the emerging clinical manifestations following NGT insertion

Nejo et al. reported that patients exhibit varying degrees of clinical signs, including hyperthermia, profuse sweating, abdominal distension and shock, upon the initiation of enteral nutrition.^54^ Given the upper gastrointestinal haemorrhage observed in our patient after NGT insertion, these emerging clinical manifestations serve as an additional warning to health care professionals. If the initial NGT position is not confirmed, attention should be paid to any subsequent changes in clinical signs. These changes include high fever, bleeding, intestinal distension and peritonitis. These symptoms could indicate additional conditions potentially related to NGT. In such cases, it is not only necessary to confirm the NGT position using a different approach but also conduct more frequent checks beyond the initial confirmation.

### 3.3 Record whether the patient has a gastrointestinal lesion

Because of the rarity of similar cases in the ICU in which the NGTs are inserted into the submucosa, we conducted a thorough review of two available reports in the literature.^54^ We discovered that one patient had already been using an indwelling NGT for an extended period, which may have caused gastrointestinal mucosal damage. In our case, the patient experienced inflammation in the lower oesophagus near the cardia. The NGT was subsequently repositioned multiple times within a short period, leading to inevitable submucosal perforation damage to the fragile cardia. Therefore, because of the severity and complexity of an ICU patient's condition, it is sometimes easy to ignore the patient's previous gastrointestinal disease because the NGT insertion is more urgent in ICU, resulting in complications such as gastrointestinal bleeding. Focusing on the patient's medical history of upper gastrointestinal diseases is also necessary.

### 3.4 Other possible reason for submucosal perforation damage

As mentioned earlier, we replaced the polyvinyl chloride NGT with polyurethane because of its superior strength, resistance to kinking and reduced risk of fracture.^63^ The use of such tubes and repeated external forces might increase the risk of tissue damage. Further research is needed to explore whether such damage is related to the NGT material.

Given the complexities of ICU patients requiring NGT, we propose several recommendations. First, contraindications to NGT should be clearly defined before NGT insertion, and special attention should be paid to verifying whether the patient has an oesophageal or gastric mucosal disease. Second, insertion should be performed gently to avoid complications in cases of resistance. Third, basic vital signs, gastrointestinal decompression, drainage colour and haemoglobin changes should be closely monitored after NGT insertion. Finally, the methods to confirm the NGT tip position are not completely safe and, in rare cases, may inadvertently tunnel under the mucosa. Therefore, other verification methods must be used. These include not only frequent pH testing combined with radiography, as recommended by existing guidelines, but also abdominal ultrasound or CT examination, especially in patients with gastrointestinal system pathologies.

## 4 CONCLUSIONS

Currently, various examination methods for determining the location of NGT have certain limitations; considering the unique disease characteristics of different patients, two or more recommended validation methods should be employed to prevent the occurrence of most complications, although some rare complications may still occur. For patients with oesophageal and gastric mucosal lesions, special attention should be paid to ensuring a smooth intubation process. Continuous monitoring of signs and symptoms suggestive of gastrointestinal injury is essential to promptly identify and manage any NGT placement‐related complications.

## AUTHOR CONTRIBUTIONS

Yiqi Zhang and Xia Zheng contributed to the conception/design of the research. Yuzhi Gao and Linyan Zeng contributed to the acquisition, analysis or interpretation of the data. Yiqi Zhang drafted the manuscript. Xia Zheng and Juan Hu critically revised the manuscript, and all authors approved the final manuscript and agree to be fully accountable for ensuring the integrity and accuracy of the work.

## ETHICS STATEMENT

This case study was approved by the clinical research ethics committee of The First Affiliated Hospital, Zhejiang University School of Medicine (No. IIT20240936A).

## PATIENT CONSENT STATEMENT

The authors declare to have the written informed consent of the son of the patient involved in the reported case to prepare and publish the case report. The patient himself could not give his consent for obvious reasons.

## Data Availability

The data that support the findings of this study are available from the corresponding author upon reasonable request.
